# The Association Between Maternal Body Fat Percentage and the Risk of Gestational Diabetes Mellitus

**DOI:** 10.7759/cureus.74125

**Published:** 2024-11-20

**Authors:** Poramed Tunkemrat, Prasert Sunsaneevithayakul, Dittakarn Boriboonhirunsarn

**Affiliations:** 1 Obstetrics and Gynecology, Faculty of Medicine Siriraj Hospital, Bangkok, THA

**Keywords:** body composition, body fat percentage, body mass index, diagnosis, gestational diabetes mellitus

## Abstract

Objective

In this study, we aimed to evaluate the relationship between body fat percentage (BFP) and the risk of gestational diabetes mellitus (GDM).

Methods

We conducted a cohort study involving 336 singleton pregnant women attending an antenatal care clinic before 14 weeks of gestation. Body composition was measured during their first antenatal visit by using a multi-frequency segmental body composition analyzer. GDM was diagnosed by a 50-g glucose challenge test (GCT) and a 100-g oral glucose tolerance test (OGTT) during the first visit and repeated during 24-28 weeks of gestation. Rates of GDM were compared between women with BFP ≥30% and those with BFP <30%. The ability of BFP and body mass index (BMI) to diagnose GDM was assessed, as well as their correlation.

Results

Of the 296 women included in the analysis, 171 had BFP ≥30%, and 125 had BFP <30%. The prevalence of GDM was 17.9%. BFP correlated well with BMI (correlation coefficient: 0.956, p<0.001). BFP ≥30% and BMI ≥25 kg/m^2^ significantly increased the risk of GDM (22.2% vs. 12%, p=0.023 and 26.4% vs. 14.4%, p=0.014, respectively). The sensitivity of BFP ≥30% and BMI ≥25 kg/m^2^ for GDM diagnosis was 71.1% and 43.3%, respectively while the specificity was 45.3% and 73.7%, respectively. Both BFP and BMI had comparable efficacy in diagnosing GDM [areas under the receiver operating characteristic (ROC) curves (AUC) of 0.634 and 0.642, respectively].

Conclusions

BFP ≥30% and BMI ≥25 kg/m^2^ significantly increased the risk of GDM. BFP correlated well with BMI and had similar efficacy in diagnosing GDM.

## Introduction

Gestational diabetes mellitus (GDM) is one of the most common complications during pregnancy, and it varies in prevalence depending on the population, diagnostic standards, ethnicity, and geographic region. GDM is on the rise globally, most likely due to an increase in maternal obesity [1-4]. GDM has been associated with various adverse pregnancy outcomes, including preeclampsia, cesarean delivery, shoulder dystocia, large for gestational age, macrosomia, and neonatal hypoglycemia [1-4]. Being overweight or obese is among the key risk factors for GDM, and several studies have consistently reported this association [1-6].

While body mass index (BMI) is a popular parameter for assessing overweight and obesity as well as associated pregnancy problems such as GDM, it may not accurately represent the composition and distribution of fat in all pregnant women. Previous studies have shown that BMI may not be a reliable indicator of predicting the risk of GDM since it does not account for the entire range of body fat mass and distribution. On the other hand, maternal central obesity may be a more accurate indicator of fat distribution and a significant risk factor for GDM [7-9].

Body composition measurement with the use of a multi-frequency segmental body composition analyzer by bioelectrical impedance analysis (BIA) has been widely employed in various disciplines, including obstetrics; it is considered safe during pregnancy, as reported by previous studies [10-15]. Among body composition measurements, fat mass has been shown to be a significant risk factor for several pregnancy-related issues, such as GDM and preeclampsia [10-15]. Some studies have reported that the risk of GDM increased by 1.3-1.8 folds with increased body fat percentage (BFP) [12-14]. One study has reported that pregnant women with a BFP ≥28% during early pregnancy have a 1.6-fold higher risk of GDM [12]. In addition, fat mass has been reported to be positively associated with birth weight as well [16].

The use of BFP might be valuable in predicting GDM as it might better reflect body fat compared to BMI. However, there is currently scarce data on the use of BFP in Siriraj Hospital and in Thailand, especially in the prediction of GDM. Therefore, the primary objective of this study was to assess the relationship between BFP and the risk of GDM. Additionally, it aimed to evaluate the diagnostic ability of both BMI and BFP.

## Materials and methods

After obtaining approval from the Siriraj Institutional Review Board (SIRB), a prospective cohort study was conducted at the Department of Obstetrics and Gynecology, Faculty of Medicine Siriraj Hospital from April 1, 2021, to March 31, 2022. Informed content was obtained from all participants. Singleton pregnant women who were at least 18 years old, visited an antenatal clinic before 14 weeks of gestation, and were diagnosed with gestational diabetes mellitus per institutional guidelines were included. Women with pre-pregnancy diabetes, incomplete screening tests, fetal deaths or anomalies, and those with electric or metal devices or any electronic medical implant in the body were excluded. The sample size was calculated based on a pilot study that reported the rates of GDM as 25% and 10% in women with BFP ≥30% and <30%, respectively. At 95% significance level and 80% power, the sample size required was 122 women in each group.

Body compositions of each woman were measured during their first antenatal visit, using a multi-frequency segmental body composition analyzer (TANITA® BC-120, Tanita Corporation of America, Inc., Arlington Heights, IL). For body composition measurement, pregnant women were checked to ensure they had not had strenuous physical activity in the previous 12 hours and asked to urinate and remove any metal jewelry (e.g., rings, wristwatches, necklaces) before the measurement. During the measurement, pregnant women were asked to remove their socks and shoes before placing both feet in the designated positions on the scale. The cohort was classified into two groups - the study group: women with BFP ≥30%; and the comparison group: those with BFP <30%.

All women received GDM screening with a 50-g glucose challenge test (GCT), and a 100-g oral glucose tolerance test (OGTT) was used to diagnose GDM during their first visit and repeated during 24-28 weeks of gestation. If the women had a 50-g GCT result of ≥140 mg/dL, a 100-g OGTT was offered based on Carpenter and Coustan criteria to diagnose GDM. All women received standard care during pregnancy until delivery.

The collected data included baseline characteristics, GDM risk factors, obstetric data, antenatal care data, results of body composition measurements, and screening and diagnosis of GDM at the first antenatal care visit and 24-28 weeks of gestation.

Descriptive statistics were used to describe various characteristics using mean, standard deviation (SD), number, and percentage, as appropriate. Comparisons between groups were performed using student t-test and chi-square test. Rates of GDM were compared between women with BFP ≥30% and those with BFP <30%. The correlation between BFP and BMI was evaluated and the correlation coefficient was estimated. The diagnostic ability of BFP and BMI for GDM was assessed using areas under the receiver operating characteristic (ROC) curves (AUC). A p-value <0.05 was considered statistically significant.

## Results

A total of 336 pregnant women who completed the body composition evaluation were initially included; 40 were excluded from the analysis: 11 women had fetal deaths, nine did not have 100-g OGTT after initial tests, and another 20 did not have 100-g OGTT during repeated tests. Finally, 296 women were included in the final analysis. Of them, 171 had BFP ≥30%, and 125 had BFP <30%.

**Figure 1:**
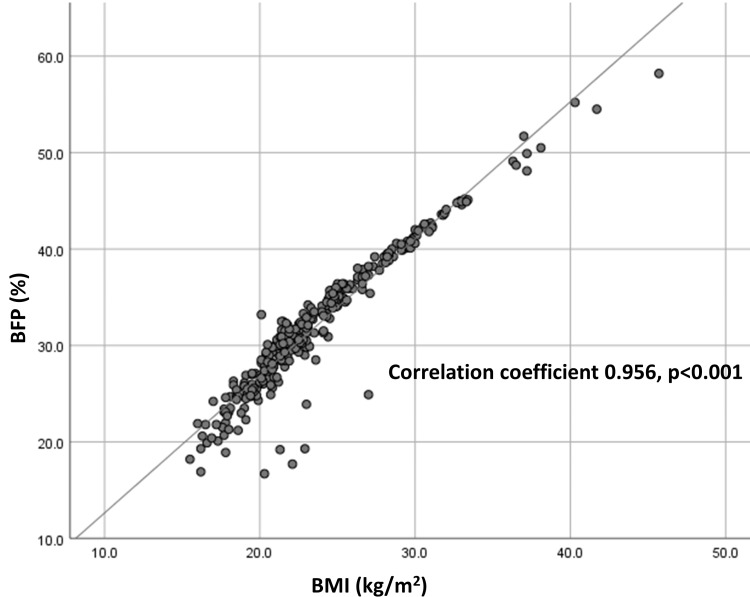
Scatterplot diagram depicting BFP and BMI BFP: body fat percentage; BMI: body mass index

Comparisons of baseline characteristics between the two groups are presented in Table 1. Women with BFP ≥30% were significantly older (mean age: 30.9 ±5.5 vs. 28.7 ±5.9 years, p=0.001), had higher pre-pregnancy BMI (26.3 ±4.5 vs. 19.9 ±1.8 kg/m^2^, p<0.001), and were less likely to be nulliparous (44.4% vs. 61.6%, p=0.004). In terms of GDM risks, those with BFP ≥30% were significantly more likely to be aged ≥30 years, have DM in the family, and be overweight or obese. Of note, none with BFP ≥30% were underweight, and none with BFP <30% were overweight or obese. BFP correlated well with BMI, with a correlation coefficient of 0.956 (p<0.001) (Figure 1).

**Table 1:** Comparison of the characteristics of pregnant women between the two groups ^a^Student's t-test. ^b^Chi-square test BFP: body fat percentage; BMI: body mass index; DM: diabetes mellitus; GA: gestational age; GDM: gestational diabetes mellitus; SD: standard deviation

Characteristics	BFP <30% (n=125)	BFP ≥30% (n=171)	P-value
	Mean ±SD	Mean ±SD	
Age, years	28.7 ±5.9	30.9 ±5.5	0.001^a^
GA at measurement, weeks	8.5 ±2.3	8.6 ±2.3	0.912^a^
BMI, kg/m^2^	19.9 ±1.8	26.3 ±4.5	<0.001^a^
	N (%)	N (%)	
Nulliparity	77 (61.6)	76 (44.4)	0.004^b^
BMI category			<0.001^b^
Underweight	26 (20.8)	0 (0)	
Normal	99 (79.2)	84 (49.1)	
Overweight/obese	0 (0)	87 (50.9)	
GDM risks			
Age ≥30 years	50 (40)	104 (60.8)	<0.001^b^
DM in family	13 (10.4)	31 (18.1)	<0.001^b^
Previous GDM	0 (0)	4 (2.3)	0.141^b^
Hypertension	0 (0)	5 (2.9)	0.076^b^

Table 2 shows comparisons of various body composition measurements between the two groups. Women with BFP ≥30% had significantly higher mean fat percentage (36.5 ±5.6 vs. 25.7 ±3.2), fat mass (24.7 ±8.8 vs. 13.0 ±2.6 kg), fat-free mass (41.2 ±4.2 vs. 37.3 ±3.1 kg), muscle mass (38.8 ±3.9 vs. 35.2 ±2.9 kg), and total body water (29.5 ±3.8 vs. 25.4 ±2.2 kg), but lower total body water percentage (46.0 ±2.2 vs. 50.7 ±2.2).

**Table 2:** Comparison of body composition measurements between the two groups ^a^Student's t-test BFP: body fat percentage; SD: standard deviation

Measurements	BFP <30% (n=125)	BFP ≥30% (n=171)	P-value^a^
	Mean ±SD	Mean ±SD	
Fat percentage, %	25.7 ±3.2	36.5 ±5.6	<0.001
Fat mass, kg	13.0 ±2.6	24.7 ±8.8	<0.001
Free fat mass, kg	37.3 ±3.1	41.2 ±4.2	<0.001
Muscle mass, kg	35.2 ±2.9	38.8 ±3.9	<0.001
Total body water, kg	25.4 ±2.2	29.5 ±3.8	<0.001
Total body water percentage, %	50.7 ±2.2	46.0 ±2.2	<0.001

A total of 53 women were diagnosed with GDM, corresponding to a prevalence of 17.9%. Table 3 shows the evaluation of the risk of GDM according to BFP and BMI. BFP ≥30% and BMI ≥25 kg/m^2^ significantly increased the risk of GDM (22.2% vs. 12%, p=0.023 and 26.4% vs. 14.4%, p=0.014, respectively). The sensitivity of BFP ≥30% and BMI ≥25 kg/m^2^ for GDM diagnosis was 71.1% and 43.3%, respectively, while the specificity was 45.3% and 73.7%, respectively.

**Table 3:** Risk of GDM according to BFP and BMI ^a^Chi-square test For BMI ≥25 kg/m^2^: sensitivity: 43.3%, specificity: 73.7%. For BFP ≥30%: sensitivity: 71.7%, specificity: 45.3% BFP: body fat percentage; BMI: body mass index; GDM: gestational diabetes mellitus

Variables	No GDM (n=243), n (%)	GDM (n=53), n (%)	P-value
BFP, %			0.023^a^
<30% (n=125)	110 (88%)	15 (12%)	
≥30% (n=171)	133 (77.8%)	38 (22.2%)	
BMI, kg/m^2^			0.014^a^
<25 kg/m^2^ (n=209)	179 (85.6%)	30 (14.4%)	
≥25 kg/m^2^ (n=87)	64 (73.6%)	23 (26.4%)	

**Figure 2:**
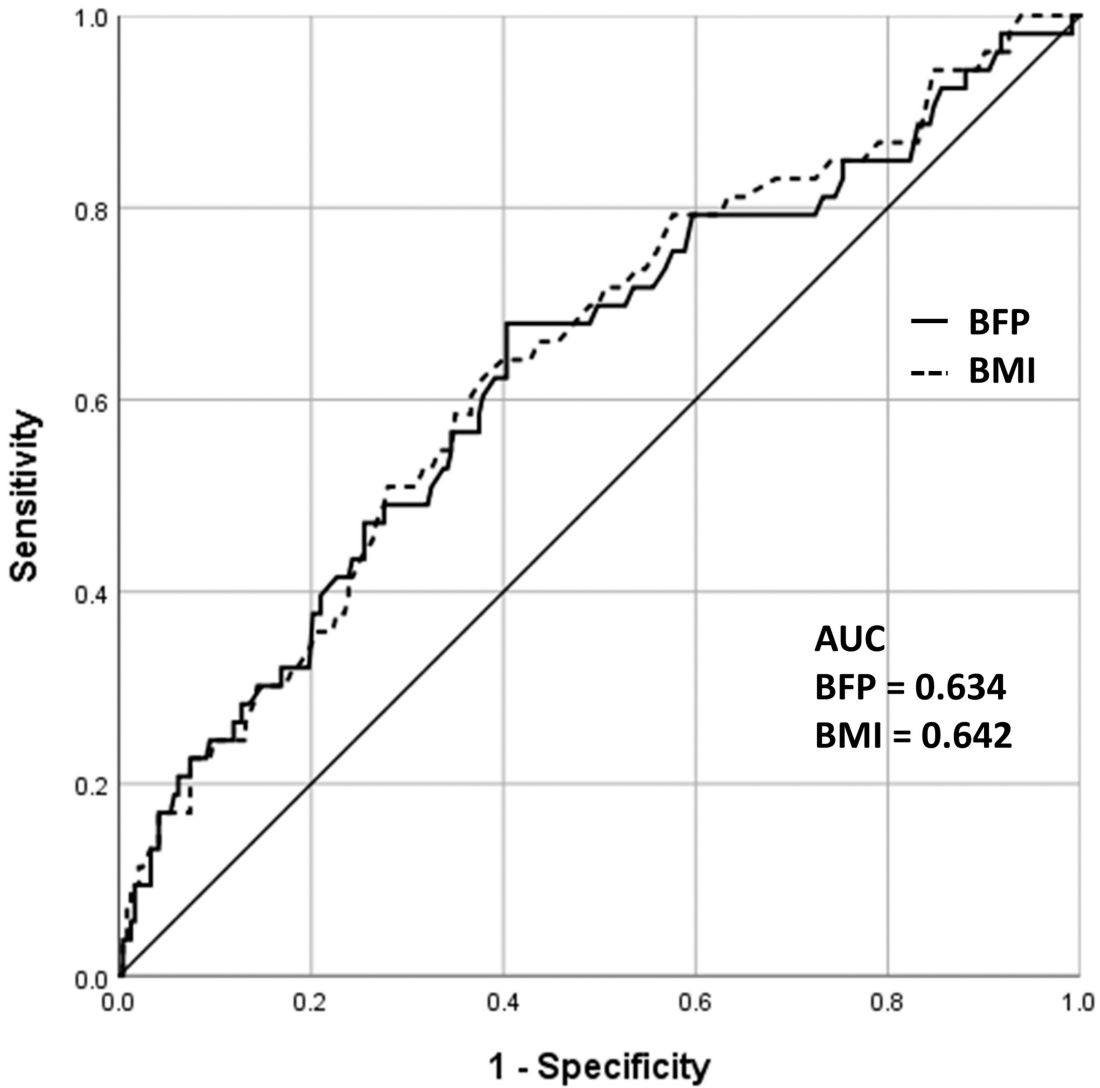
ROC curve of BFP and BMI for GDM diagnosis AUC: area under the ROC curve; BFP: body fat percentage; BMI: body mass index; GDM: gestational diabetes mellitus; ROC: receiver operating characteristic

The diagnostic ability of BFP and BMI for GDM was evaluated by ROC curve analysis and the results are shown in Figure 2. AUCs were comparable between BFP and BMI (0.634 and 0.642, respectively).

## Discussion

Even though BMI is a widely used indicator of obesity, it cannot provide information regarding the composition and distribution of body fat. According to a previous systematic review, maternal central obesity would be more accurately reflective of fat distribution and could be a significant risk factor for GDM [8]. Among other methods for assessing body composition in pregnancy, BIA provides reliable estimations of body composition early in pregnancy [17,18]. Since it is affordable and practical, BIA is used extensively across a wide range of disciplines and scenarios. It has been reported that BIA can be used to evaluate a pregnant woman's fat mass, which can be considered an important risk for various pregnancy complications, including GDM and preeclampsia [10-15].

Our results showed that the GDM risk significantly increased in women with BFP ≥30%, which aligns with the findings of other studies. A previous study evaluated body composition during early pregnancy at 13 weeks of gestation and showed that BFP was significantly higher in GDM while skeletal muscle mass percentage was lower. In addition, women with BFP ≥28% were at higher risk for GDM [12]. Another study reported that a BFP of >25% during the second trimester was the strongest risk factor for GDM [14]. Hyperglycemia in pregnancy has also been reported to be significantly associated with BFP in another study [13]. These findings are consistent with prior research, which found that visceral and subcutaneous fat evaluated via abdominal ultrasound predicted the development of GDM [19-23]. The higher risk of GDM among those with high BFP might be due to an increase in insulin resistance, as reported previously [24,25].

High BMI has been generally accepted as a significant risk for GDM [1-3]. Our findings showed that being overweight or obese, as measured by BMI, significantly increased the risk of GDM. Moreover, BMI and BFP correlated well, with a correlation coefficient of 0.956; none of the underweight women in our cohort had BFP ≥30% and all of the overweight and obese women had BFP ≥30%. In terms of diagnostic ability of BFP and BMI, the use of BFP ≥30% and BMI ≥25 kg/m^2^ for GDM diagnosis had a sensitivity of 71.1% and 43.3% respectively, and a specificity of 45.3% and 73.7%, respectively. Further analysis showed that AUCs were comparable between BFP and BMI (0.634 and 0.642, respectively). This is similar to the results from a previous study [12]. However, another study reported that BFP might be of more value than BMI as it represents the strongest risk for GDM [14].

The use of BFP as a measurement of obesity and a tool to stratify the risk of adverse outcomes might provide more accurate results than the use of BMI. A previous study showed that as many as 22% of the women were classified incorrectly based on self-reported BMI; 12% of the women were classified as having a normal BMI while they were actually overweight and 5% of those classified as overweight were obese [26]. The differences in results across studies could be due to differences in population characteristics and GDM risks, timing of body composition measurements, and screening and diagnostic protocols for GDM. However, the results more or less pointed to a similar trend, suggesting that BFP measurement in either the first or second trimester of pregnancy might be of value in GDM prediction.

Strengths and limitations

The strengths of this study include the unbiased measurement of BFP and other body compositions. Early measurements could reflect the pre-pregnancy status of the women, as body composition values have been reported to remain unchanged in the first trimester of pregnancy [27]. In addition, GDM screening and diagnosis were uniform, and consistent with the institutional protocol. However, the study has some limitations. Some women were excluded from the analysis due to various reasons, which might affect the results. However, the remaining samples had similar characteristics and prevalence of GDM to those of women attending antenatal care, and hence we believe the samples are still representative. Generalization of the results might be restricted due to differences in demographic characteristics and GDM screening and diagnostic protocols. Further research with larger samples and more detailed analyses is required to gain deeper insights into the value of body composition measurements in predicting GDM as well as other complications.

## Conclusions

Our findings showed that BFP ≥30% during the first trimester significantly increased the risk of GDM. BFP correlated well with BMI and showed comparable diagnostic ability for GDM. BIA is a rapid, noninvasive, valid, inexpensive, and simple method for body composition measurements, and we recommend its wide use as a risk stratification tool in addition to BMI.

## Figures and Tables

**Figure 3:**
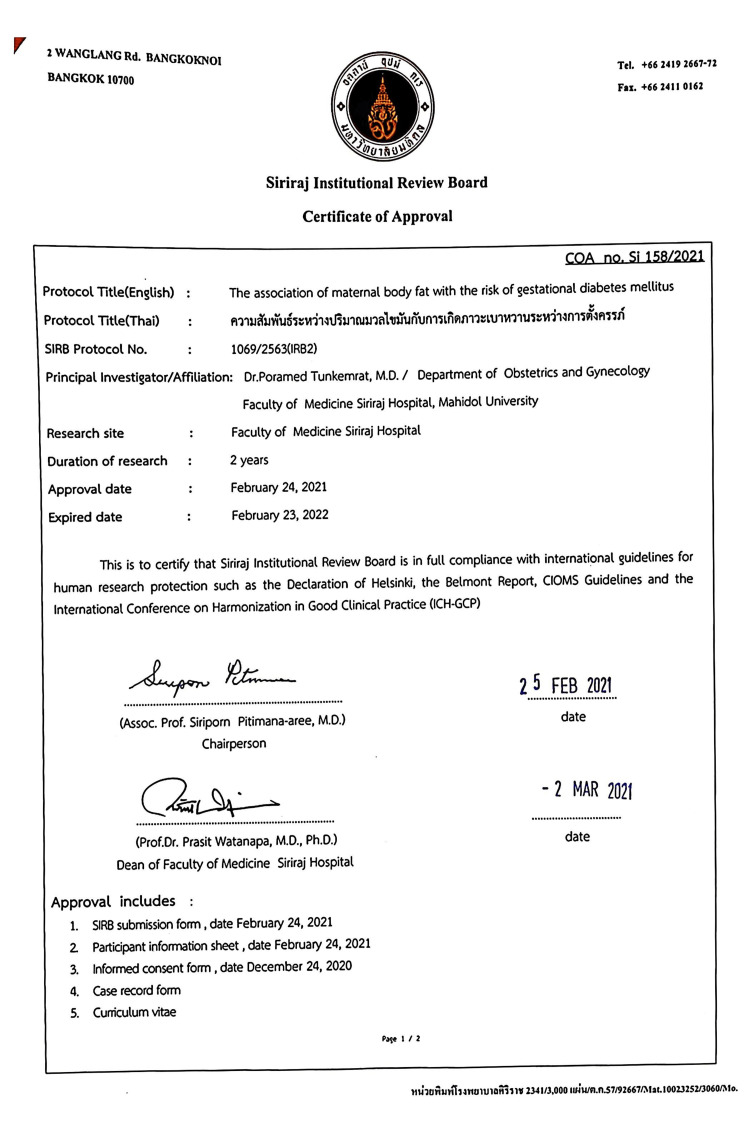
Institutional Review Board (IRB) approval letter

## Appendices

Letter of approval from Siriraj Institutional Review Board
